# Differences between inflamed and non inflamed appendices diagnosed as acute appendicitis

**DOI:** 10.1016/j.amsu.2021.01.044

**Published:** 2021-01-18

**Authors:** Pedro Luiz do Nascimento Junior, Carlos Teixeira Brandt, Andy Petroianu

**Affiliations:** University of Social and Applied Sciences, Campina Grande City, Brazil

**Keywords:** Acute appendicitis, Noninflammatory appendiceal disease, Appendectomy, Cecal appendix, Diagnosis, Treatment

## Abstract

**Background:**

Despite the great advances in diagnostic methods, the incidence of the surgical removal of a morphologically normal appendix in patients with clinical and complementary signs of acute appendicitis continues to exceed 20%. This study aimed to compare the clinical, laboratory, and ultrasound findings of inflammatory and noninflammatory appendiceal disorders diagnosed as acute appendicitis.

**Methods:**

The medical records of 208 patients with clinical, laboratory, and ultrasound findings indicative of acute appendicitis were studied. The patients were divided into two groups: group 1 comprising 94 patients whose appendicular histological results suggested a normal appendix and group 2 comprising 114 patients with histopathological tests confirming acute appendicitis. The variables analyzed were age at the time of surgery, sex, nausea and vomiting, inappetence, fever, pain migrating to the right iliac fossa, pain on palpation of the right iliac fossa, Blumberg's sign, blood counts, ultrasound findings, and Alvarado score.

**Results:**

An inflamed appendix was associated with inappetence, pain on palpation of the right iliac fossa, appendiceal diameter >6 mm, and Alvarado score >6 (p < 0.001). In contrast, fever was more frequently found in noninflammatory appendiceal disorders (p < 0.001).

**Conclusion:**

Inappetence, pain on palpation of the right iliac fossa, appendiceal diameter > 6 mm, and Alvarado score > 6 indicate an inflammatory appendiceal disease, whereas fever is more often present in noninflammatory appendiceal diseases.

## Highlights

• Morphologically normal appendices are found in more than 20% of appendectomies.
• All manifestations of acute appendicitis disappear immediately after appendectomy even when the appendix seems to be normal.
• Classical pathophysiology theories attributed to acute appendicitis cannot be proved in experimental and clinical studies.
• Non inflammatory appendiceal disorders may be associated with similar manifestations of acute appendicitis.
• Specific manifestations of acute appendicitis may be different in inflammatory and not inflammatory appendices.

## 1 Introduction

Acute appendicitis is one of the most frequent causes of an acute surgical abdomen. It has an incidence of approximately 233 in 100,000 people, being more common in men (1.4:1), and occurs throughout life in 8.6% of men and 6.7% of women [[1], [2], [3]]. Most patients with appendicitis are white-skinned (74%), with a low incidence in black-skinned people (<5%) [[3], [4], [5]]. Annually, approximately 310,000 appendectomies are performed in the United States and 47,000 in the United Kingdom, at a mean cost of US $ 33,000 per patient in the United States [[6], [7], [8]]. Studies in the United Kingdom have indicated that more than one-fifth of the appendectomies show normal histopathological results [9]. Cases from other countries have also presented normal histological results in >20% of the cases with typical clinical and complementary signs of acute appendicitis and with surgical indications owing to diagnostic uncertainty [10].

Although the diagnosis of appendicitis is not challenging, atypical presentations can result in inappropriate management [11,12]. Lu et al. (2016) reported that appendectomies indicated for uncertain cases are associated with postoperative complications, mainly local abscess, and adhesion-related chronic pain [13].

The most accepted pathophysiological theory for acute appendicitis is appendiceal obstruction by fecaliths, foreign bodies, seeds, parasites, lymphoid hyperplasia, infectious processes, and benign or malignant tumors, which increase the intraluminal and intramural pressures, resulting in thrombosis, and blood and lymphatic vascular occlusion [1,11]. As blood and lymphatic vascular involvement progress, stasis occurs and the appendiceal wall becomes ischemic and necrotic [11].

However, intraluminal appendiceal content is often found in normal appendices without causing inflammation, appendiceal hypertension, or any other signs [1,11]. Therefore, the etiopathogenesis of acute appendicitis remains unknown. Moreover, many of the theories are not supported by clinical, complementary, and histological tests, and do not explain the follow-up of many patients [14,15]. It has not yet been possible to cause experimental acute appendicitis that is morphologically similar to human appendicitis to prove the pathophysiological theories. Multiple appendiceal diseases with clinical and complementary conditions similar to those of acute appendicitis but without appendiceal inflammation and with possible neurogenic, endocrine, or immune causes must be considered [16,17].

Although the appendix appears as a projection of the cecal wall, its morphological characteristics differ from those of the rest of the digestive tract. The appendix has a much larger number of lymphatic follicles and cells of the neuroendocrine system in the Lieberkühn crypts, indicating the relationship of this organ to the immune and neuroendocrine systems [[18], [19], [20], [21], [22], [23], [24]]. Although the most common histological type of digestive system malignancies is adenocarcinoma, the prevalent cecal appendiceal neoplasm is a carcinoid tumor, which belongs to the neuroendocrine system and is found in up to 0.7% of all appendectomies [19].

Apparently normal appendices removed from patients with a clinical presentation of acute appendicitis present changes in substance P neuroendocrine markers, vasoactive intestinal polypeptide, gastric inhibitory polypeptide, calcium-binding protein, cyclooxygenases 1 and 2, tumor necrosis factor, prostaglandin E2, mast cell tryptase, nitric oxide synthase, CD8 lymphocytes, protein gene product 9.5, vascular endothelial growth factor, class 2 histocompatibility complex, synaptophysin, enolase, and S100 protein [[20], [21], [22], [23], [24]]. Changes in these neuroimmune modulators have been proven in the presence of a clinical presentation of acute appendicitis without appendiceal inflammation, indicating the possibility of a neuroimmune endocrine appendiceal disease simulating acute appendicitis [22,23].

The clinical presentation of both acute appendicitis and neuroendocrine appendiceal disease includes migrating abdominal pain from the upper abdomen to the periumbilical region and subsequently to the right lower quadrant, as well as inappetence, nausea and vomiting associated with dyspepsia, and evacuation changes. About 80% of the patients have leukocytosis and left shift [22,23,25]. Cecal dilation with fecal content inside the cecum is identified in 98% of the patients with acute appendicitis [14,15]. Ultrasound (US) and computed tomography (CT) show cecal dilation with fecal content as well as appendiceal wall thickening > 2 mm, appendiceal diameter > 6 mm, and enlarged peri-appendiceal connective tissues [[12], [13], [14], [15]].

Several scoring systems have been created for greater diagnostic accuracy [[26], [27], [28], [29], [30], [31], [32], [33]]. The most frequently used is the Alvarado score, with a score >7 indicating an increased probability of acute appendicitis. Another scoring system is the appendicitis inflammatory response (AIR), which uses clinical criteria such as vomiting, fever, and pain intensity in the right iliac fossa (RIF) associated with leukocyte count and *C*-reactive protein (CRP) level to quantify the intensity of the inflammatory response [28]. Another score is the adult appendicitis score (AAS), which uses clinical data, white cell count, and CRP level. These criteria have been reported to reduce the incidence of the surgical removal of apparently normal appendices to 66%, compared with cases in which they were not used [33]. A radiographic score has also been developed at a hospital in Singapore, the Raja Isteri Pengiran Anak Saleha Appendicitis (RIPASA) score, with greater sensitivity and specificity in Eastern populations than the Alvarado score [29,30]; however, its effectiveness in Western populations is yet to be proven [31].

Even when all these scores are correctly used, the possibility of diagnostic errors and delayed proper management persist [27]. None of these scores consider ethnic group diversity and the patients’ age and sex. Especially, they do not consider that the prevalent age group for acute appendicitis is the same as that for female pelvic inflammatory diseases.

The high diagnostic uncertainty based on clinical and complementary tests prompted this study, which aimed to investigate the parameters used in the diagnosis of acute appendicitis and to verify the existence of differences concerning noninflammatory appendiceal diseases with similar clinical and complementary presentations. Thus, this study, which belong to a line of research, aimed to compare the clinical, laboratory and US findings between inflammatory and noninflammatory appendiceal disorders diagnosed as acute appendicitis [3,12,14,15,22,23,46,47].

## 2 Methods

This study met the requirements of the Declaration of Helsinki for research involving human subjects and Resolution 466/2012 of the National Health Council (Brazilian Ministry of Health 2012). Data were collected after obtaining approval from the Research Ethics Committee of the Research Ethics Committee of the University of Social and Applied Sciences (3894634). This work has been reported in line with the STROCSS criteria [34].

This retrospective study analyzed the medical records of 208 patients with clinical, laboratory, and US findings indicative of acute appendicitis at the Campina Grande Emergency and Trauma Hospital between 2010 and 2019. The histological findings of the surgically removed appendices were analyzed, with a focus on inflammatory signs, and the patients were divided into two groups:

- group 1–94 patients with noninflamed appendices and
- group 2–114 patients with histopathological tests confirming acute appendicitis.

The analyzed variables were age at the time of surgery, sex, nausea and vomiting, inappetence, fever, pain migrating to the RIF, pain on palpation of the RIF, Blumberg's sign, leukocytosis (white blood cells > 10,000/mm^3^) and left shift, appendiceal diameter > 6 mm on US, and Alvarado score.

Statistical analyses were performed using IBM SPSS Statistics software, version 20.0. Qualitative variables are expressed as absolute and relative frequencies, and quantitative variables are expressed as mean and standard deviation of the mean. Categorical variables were compared in contingency tables using chi-square tests of associations. The Mann-Whitney test was used to analyze age and the Alvarado score. The Student's t-test was used for dichotomous variables. Association measures were used for qualitative variable associations; however, as this was a cohort study, the relative risk was applied. The level of significance was set at > 95%, corresponding to a p < 0.05.

## 3 Results

Group 1 included 44 men (46.8%) and 50 women (53.2%) and group 2 included 55 men (48.2%) and 59 women (51.8%), indicating sex distribution homogeneity (p = 0.836). The patients’ ages ranged from 10 to 86 years (32.7 ± 15.1 years) in group 1 and from 16 to 79 years (33.5 ± 15.8 years) in group 2, with no difference between groups (p = 0.684).

Inappetence, pain on palpation of the RIF, fever, and appendiceal diameter >6 mm were found to be associated with inflamed appendices. The other variables showed no differences. In contrast, fever was more often associated with noninflammatory appendiceal diseases (Table 1). Most patients with an inflamed appendix had inappetence, whereas only one-third of the patients with a non inflamed appendix had this symptom. Almost all patients with appendiceal inflammation reported pain on palpation of the RIF, whereas only half of the patients without inflammation reported this symptom. Fever was present in less than a quarter of the inflamed appendices and in almost three quarters of the non inflamed appendices.

**Table 1:** Clinical, laboratory, ultrasound, and Alvarado score evaluation in patients with clinical and complementary symptoms of acute appendicitis with morphologically normal (group 1) and inflamed (group 2) appendices.

Variables	Group 1 n (%)	Group 2 n (%)	p	Relative risk
Pain migrating to the RIF	77 (81.9)	96 (84.2)	0.685*	1.06
Inappetence	36 (38.3)	83 (72.8)	0.027*	1.29
Nausea and vomiting	69 (73.4)	86 (75.4)	0.738*	1.050
Fever	61 (64.9)	27 (23.6)	0.001*	**2.52**
Pain on palpation of the RIF	51 (54.6)	100 (87.7)	0.001*	**2.696**
Positive Blumberg's sign	68 (77.1)	88 (77.1)	0.421*	1.128
Leukocytes > 10,000/mm^3^	54 (57.4)	68 (59.6)	0.748*	1.042
Left shift	17 (18)	24 (21)	0.592*	1.086
Appendiceal diameter > 6 mm (US)	25 (26.6)	91 (79.8%)	0.001*	**3.138**
Alvarado score (M ± SDM)	3.34 ± 2.95	6.29 ± 1.88	0.001**	**-**

n, absolute number; RIF, right iliac fossa; M, mean; SDM, standard deviation of the mean; p, significance value; *chi-square test; **Mann-Whitney test.

An appendiceal diameter >6 mm on US was found in more than three-quarters of the inflamed cases and only a quarter of the noninflamed cases. Alvarado score >6 was found in patients with inflammatory appendiceal diseases and <4 in those with noninflammatory appendiceal diseases (Table 1).

## 4 Discussion

In many cases of acute appendicitis diagnosed based on the clinical presentation and complementary tests, the intraoperative morphological appearance and pathological findings show no appendiceal inflammation. This appendiceal disease has clinical presentations and laboratory and imaging test results similar to those of acute appendicitis, and also presents histologically increased lymph and nervous tissues, especially in the submucosa [16,17,[20], [21], [22], [23], [24]].

Notably, some patients diagnosed with acute appendicitis show complete and definitive healing with antibiotic treatment alone [35,36]. In contrast, others temporarily improve. However, the disease worsens after treatment, progressing to complicated acute appendicitis [[37], [38], [39]]. These varied results of clinical treatment may be due to different appendiceal diseases. Clinical treatment for an uncertain type of appendiceal disease is only indicated when there is a hindrance to performing an appendectomy, such as the absence of a surgeon or an adequate surgical environment. As the state of the appendix and the type of appendiceal disease that responds to antibiotic therapy are unknown, it is not safe to indicate clinical treatment for acute appendicitis.

The removal of an apparently normal appendix in patients with clinical and laboratory findings indicative of acute appendicitis is associated with an immediate and definitive cessation of clinical complaints and signs found in complementary laboratory and radiological tests [40]. This postoperative progression suggests that the apparently normal appendix removed due to clinical conditions indicative of acute appendicitis should have been a noninflammatory disease that was not diagnosed on routine histological examination. If the acute abdomen was not caused by an appendiceal disease, immediate improvement soon after the removal of the appendix would not occur [41].

Elderly and immunosuppressed patients present clinical signs of acute appendicitis associated with ischemia and increased D-dimer levels [42]. These patients probably have no appendiceal inflammation and their ischemia is caused by a vascular obstruction. This may be the reason why appendicitis in these patients rapidly progresses to generalized peritonitis without appendiceal obstruction by peritoneal structures characteristic of acute appendicitis. However, this study showed no differences in results in terms of patient age and gender.

Even without using radiological criteria, the Alvarado, AAS, and AIR scores are associated with a lower incidence of normal appendices than cases in which these scores were not used [[43], [44], [45], [46]]. This study also shows the importance of an Alvarado score >6, which is associated with appendiceal inflammation.

Appendiceal luminal dilation and wall thickening, as well as cecal dilation with fecal content are signs of an inflammatory appendiceal disorder with a specificity superior to 90% [14,15]. Operations performed on patients without radiological specific signs of acute appendicitis are associated with a higher incidence of non inflamed appendices. This association was confirmed in this study when appendices with a diameter >6 mm, which were mostly found in inflamed appendices.

Noninflammatory neuroimmune-endocrine appendiceal disorders can only be identified using specific immunohistochemical tests for specific mediators present in the surgically removed appendices [19]. Barroso and Petroianu conducted an extensive literature review and found 14 neuropeptides confirming the existence of a neurogenic disease in morphologically normal appendices surgically removed from patients with clinical presentations of acute appendicitis [22,23]. In 2020, these authors studied 12 neuroimmune-endocrine mediators in normal appendices of patients without appendiceal complaints, in apparently normal appendices of patients with a clinical presentation of acute appendicitis, and in non inflamed appendices. Morphologically normal appendices removed owing to a clinical diagnosis of appendicitis were associated with increased expression of neuroimmune-endocrine mediators, with emphasis on synaptophysin, enolase, mast cell-related tryptase, and protein gene product 9.5 in the appendiceal wall [47]. Therefore, clinical, laboratory and imaging findings characteristic of acute appendicitis may be associated not only with inflammatory but also with neuroimmune-endocrine disorders.

This study found that inappetence, pain on palpation of the RIF, appendiceal diameter >6 mm on US, and Alvarado score >6 indicate inflammatory appendicitis. In contrast, fever was more frequent in noninflammatory appendiceal disorders. Although fever is characteristic of infectious diseases, several peptides, such as interleukins 1 and 6 and tumor necrosis factor, also act on the posterior hypothalamus, inducing prostaglandin production in the hypothalamic endothelium and pineal region, activated by the systemic release of peptides from the appendix, resulting in increased temperature as they act on the hypothalamic temperature-regulating center [[48], [49], [50], [51], [52]].

Inappetence is the most frequent symptom of all inflammatory disorders, to the extent that its absence in acute appendicitis leads to diagnostic uncertainty, considering the frequency of this symptom in many acute and chronic disorders [53]. Similar to fever, inappetence results from changes in the activation of prostaglandins and other mediators in the lateral hypothalamus and other areas of the central nervous system [54,55].

Although the value of the Alvarado score is highly questioned, this study showed that an Alvarado score >6 has a high specificity for inflammatory appendiceal diseases. However, the limitation of this score is the absence of imaging tests, which, as this study showed, are the most sensitive diagnostic tools for identifying an inflamed appendix. Therefore, the Alvarado score should be associated with radiographic, US, or CT imaging examinations to increase its specificity [12,14,15,[44], [45], [46]].

The comparison between inflammatory and noninflammatory appendiceal diseases showed that pain is more often present when the appendix is inflamed. Pain has been known as a characteristic of all inflammatory conditions since Galen's writings and probably even long before his time. However, neuroendocrine mediator disorders can also cause pain, even if it is less intense and not caused by peritonitis.

The main limitations of this study were due to its retrospective characteristics. This investigation had to be restricted to the data that were found in all charts in order to perform a correct statistical analysis. Another aspect to be considered is related to the different surgeons who handled the patients. Even being a single service with standard procedures personal approaches cannot be avoided. However, all records were precise and correct, as well as the histological study which was revised by only one pathologist with great experience in digestive diseases. This study belongs to a line of research with several previous publications [3,12,14,15,22,23,46,47]. Actually, other investigations are performed to understand the appendix and the appendicopathies. Only after a correct comprehension of this relevant organ, unnecessary appendectomies will be prevented.

## 5 Conclusion

Inappetence, pain on palpation of the RIF, appendiceal diameter >6 mm, and Alvarado score >6 indicate an inflammatory disease of the appendix, whereas fever is more often present in noninflammatory appendiceal diseases.

## Contributors

- Pedro Luiz do Nascimento Junior Acquisition of data, analysis and interpretation of data, participate in drafting the article and took responsibility for all aspects of this work and article.
- Carlos Teixeira Brandt Participate in revising critically the article and took responsibility for all aspects of this work and article.
- Andy Petroianu Conception and design the study, analysis and interpretation of data, participate in revising critically the article and took responsibility for all aspects of this work and article.

All authors accept direct responsibility for the manuscript and gave final approval of the version to be published.

## Declaration of conflicting interests

The authors declare no conflict or competing interest with respect to the research, authorship and publication of this article. The authors have no financial relationship with any organization. The authors have full control of all data, and agree to allow the journal to review any data if requested.

## Funding sources

The authors declare no funding for our research.

## Statement

Provenance and peer review. Not commissioned, externally peer-reviewed.
